# The Role of Ultrasonography in Patients Referring to the Emergency Department with Acute Abdominal Pain

**DOI:** 10.22114/ajem.v0i0.152

**Published:** 2019-05-16

**Authors:** Ali Abdolrazaghnejad, Ali Rajabpour-Sanati, Hojjat Rastegari-Najafabadi, Maryam Ziaei, Abdolghader Pakniyat

**Affiliations:** 1. Department of Emergency Medicine, Khatam-Al-Anbia Hospital, Zahedan University of Medical Sciences, Zahedan, Iran.; 2. Faculty of Medicine, Birjand University of Medical Sciences, Birjand, Iran.; 3. Department of Emergency Medicine, Faculty of Medicine, Kurdistan University of Medical Sciences, Sanandaj, Iran.

**Keywords:** Abdominal Pain, Diagnosis, Differential, Emergency Service, Hospital, Ultrasonography

## Abstract

**Context:**

Acute abdominal pain is a common clinical problem in emergency and non-emergency cases accounting for 5 to 10% of all referrals to the emergency department. Studies have indicated that these widely differentiated diagnoses are common to these complaints. Considering the high prevalence of this complaint in the patients and the wide range of its differential diagnosis, this review study was designed and evaluated aiming at investigating the causes of acute abdominal pain with a focus on assessing the position of ultrasound as a diagnostic tool in the emergency department.

**Evidence acquisition:**

This article was conducted as a narrative review of selected articles from 2005 through 2019. By comparing them, a comprehensive review of ultrasound role was conducted in patients with acute abdominal pain referring to the emergency department.

**Results:**

In this review study, we attempted to use the articles of the clinical approach, the required laboratory tests, the disadvantages and advantages of each imaging technique, the differential diagnosis for acute abdominal pain according to the location of the pain, and the position of ultrasound as a diagnostic aid tool. Eventually, the proposed protrusion will be considered in dealing with a patient with acute abdominal pain.

**Conclusion:**

Regarding the wide range of causes providing multiple differential diagnosis, as well as the limited time of the health team in the emergency department for diagnostic and therapeutic measures, particularly in time-sensitive clinical conditions, ultrasound offered by emergency medicine specialists as a diagnostic aid is considered to improve the overall diagnosis and treatment of patients, thereby reducing complications.

## Context

Acute abdominal pain is one of the most common complaint of patients referring to the emergency department (ED), accounting for 5 to 10% of all referrals to this department (1–3). The high prevalence of this complaint and its wide range of causes, from mild non-specific illness to life-threatening ones, require careful handling of such patients; as its misdiagnosis can brings devastating and also legal problems (4–6). It is obvious that timely diagnosis of the cause may lead to better management and ultimately better patients’ outcome. Identifying the cause of abdominal pain begins history taking and physical examination, empower the physician to decide the necessity of further investigations (7). Ultrasonography is one of the most prevalent test that usually requested in terms of diagnostic process of a patient with acute abdominal pain. Considering the high prevalence of this complaint and the wide range of its differential diagnosis, this review study was performed aiming at investigating the causes of acute abdominal pain with a focus on assessing the position of ultrasound (US) as a diagnostic tool in the ED.

## Evidence acquisition

This review study was conducted using PubMed, Scopus, EmBase, ScienceDirect and Google Scholar databases. Articles selected from those published from 2005 through 2019. This article was conducted as a narrative review and does not include any independent data derived from direct clinical intervention or fieldwork.

## Results

Finally, selected articles were used in writing this overview. By comparing them, it was attempted to complete a comprehensive review of ultrasonography role in patients with acute abdominal pain referring to the ED.

### Clinical evaluation of acute abdominal pain

The most important part of evaluating a patient with acute abdominal pain is history of presenting illness (6). In acute conditions, it is necessary to distinguish life-threatening conditions from non-circumstance ones. Severity determinants in abdominal pain include acute onset, increased pain, signs of peritonitis, autonomic impairment and association with gastrointestinal (GI) warning signs such as GI bleeding and unstable hemodynamics (8). In the history of presenting illness, a patient with acute abdominal pain has key characteristics that are extremely valuable in determining the exact cause. The onset time and duration of the abdominal pain in the patient may be helpful in determining the progression of the symptoms and the probability of an emergent cause. Severe abdominal pain leads to clinical suspicion of serious and emergent causes such as rupture of abdominal aortic aneurysm, mesenteric ischemia and intestinal perforation (6, 9–12). The location of abdominal pain is an important factor in determining the member involved. Identification of the embryologic derivation of the GI organs can help to limit the diagnosis to the examiner (13). Appendix 1 lists differential diagnosis abdominal pain according to the location of pain.

Abdominal pain characteristics are helpful in determining its physiopathology. The aching or dull pain is due to activation of visceral neural fibers, which may result in stretching, increased peristalsis, or ischemia. An example of activation of these nerves is colonic abdominal pain in the small bowel obstruction (14). In diseases involving the liver, biliary tract, or appendix, referral of pain can be an important and relevant factor associated with the disease. For example, gallstone pain is accompanied by a relief of pain from right upper quadrant to the back (15). The pain under the right strain is more specific for liver and bile ducts (16). In appendicitis, the sensitivity and specificity of diagnosis are indicated for the migration of pain from periumbilical to right iliac fossa (17). Coupled factors, pain-enhancing and irritating factors play a crucial role in determining the cause of pain. A patient with colicky pain usually changes his body’s condition, while the pain caused by peritonitis leads to staying motionless (18). The abdominal pain from peptic ulcer may improve with food; mesenteric ischemia can occur after eating or the symptoms of inflammatory bowel syndrome improve with fecal excretion (19, 20). Heartburn can cause a suspicion of peptic ulcer or dispositive episode (21, 22). In women of childbearing age, the history of gynecological and obstetrical diseases is necessary, since in these patients, the possibility of abdominal pain due to gynecologic problems is high (23). The type of contraceptive method, if used, is important, especially if you use tools such as intrauterine device (IUD) (24).

### Medical, pharmaceutical, and social records

Repeated pain is similar in kidney stones, diverticulitis, and gallstones, and in contrast, newborn pain can be seen in some cases such as appendicitis (25–28). The presence of comorbid diseases such as type-2 diabetes (a 1.5-fold chance for acute pancreatitis relative to non-diabetics), and atrial fibrillation (awareness of acute abdominal pain for mesenteric ischemia) can help to diagnose the causes of acute abdominal pain (29, 30). The history of drug use in identifying the cause of abdominal pain can be also helpful in cases such as an increased risk of upper GI bleeding in patients taking Nonsteroidal anti-inflammatory drugs (NSAIDs) or anticoagulants (31). The history of previous abdominal surgery increases suspicion of small bowel obstruction (32). Smoking history is associated with an increased risk of abdominal aortic aneurysm rupture (33). Injecting drug use, like cocaine, is concerned with acute ascites of ischemia (7). It is highly important in women with gynecologic history. For example, ectopic pregnancy (EP) is accompanied by previous history of IUD, history of infertility, and tubal ligation (34).

### Physical examination

After acquiring the history of presenting illness, physical examination is the next step in limiting the diagnosis. At the beginning of the physical examination, it is important and helpful to consider the patient’s general appearance, such as being comfortable or discomfortable, slimming or obesity, pallor, sweating, or sleepiness, and jaundice. A patient who is pale, confused and distressed has more likely a more severe disease. Certainly, in older patients, the patient’s response to severe illness may be less than usual. Pain induced by the slightest movement of the abdomen increases the suspicion of peritonitis, while in the patient’s renal colic, the patient is constantly moving, even while lying on the bed (2, 23, 35). Vital signs such as temperature, heart rate, breathing rate, blood pressure, and oxygen saturation are measured and recorded in each patient. Abnormality of vital signs are due to more serious causes, however, they are not normal in terms of emergent diagnosis. The presence of fever leads to more infectious or inflammatory factors, which may not be present in the elderly and immunocompromised individuals; lowering the blood pressure is suggestive of sepsis, bleeding and severe dehydration; tachycardia is suggestive of pain; sepsis; bleeding; fluid accumulation is in the third is that may not be present in patients with beta-blockers; tachypnea suggests metabolic acidosis or pain relief. It should be noted that ill patients with unstable vital signs, a series of diagnoses, including GI bleeding, ruptured abdominal aorta aneurysm, massive pulmonary embolism, perforated viscus, and ruptured EP should be considered. Signs like hypotension should be quickly compensated for by resuming the volume. In addition, the high life-threatening diseases requiring surgery should be rapidly assessed and identified. The initiation of physical examination is carried out by hand. firstly, an examination should be initiated with an inspection, which is a quick and easy way to collect information. A history of abdominal surgery present itself with surgical scarring, and it is a risk factor in cases such as intestinal obstruction and viscus perforation (32). The abdominal wall increases suspicion of diseases such as intestinal obstruction, peritoneal bleeding and ascites (14, 36). Osteomyosis suggests retroperitoneal hemorrhage. The touch of the abdomen should begin in the form of a light that can be properly evaluated for the guarding or tenderness. Deep touch begins from abdominal pain areas to examine liver, spleen, kidney, aortic aneurysm and masses. Percussion is used to confirm hepatomegaly and splenomegaly and to distinguish between distension caused by gas and fluid, which is helpful in case of abnormality of bedside ultrasonography. In the examination, it should be noted that the abdominal guards are voluntary and involuntary. Voluntary guarding can be eliminated due to fear, anxiety, ticklishness, and pressure, by distracting the patient. The involuntary or rigidity of the body represents abdominal reflexes of the abdominal muscles due to peritoneal stimulation, and its diagnostic sign is that during the respiratory cycle, there is a continuous increase in abdominal muscles. The rebound tenderness test is performed with a gentle and deep palpation seeking for a sudden examination of the fingers. Positivity of the test is to increase the pain when removing the fingers. Certainly, this test is in 0.25 patients with false positives (37). Alternatives are cough testing, jumping test, and heel-strike test (38–41). In general, the more these tests are positive, the greater the likelihood of peritonitis is. In auscultation, he should listen to the intestinal sounds for one minute. In general, it has a limited application. High–pitched sounds can exist at the early stage of the small bowel obstruction. Furthermore, frequent bowel sounds in diarrhea and gastroenteritis, and hypoactive or absent intestinal sounds can be suggestive of cases such as late bowel obstruction, and ileus (42). Important red flag marks in abdominal pain include unwanted or unexplained weight loss, rectal bleeding, familial history of intestinal or ovarian cancer, or altered bowel habits as loose or repeated stools lasting for more than six weeks in an individual over 60 years old (8). Rectal examination can be helpful in cases of constipation, anorectal complaints or GI bleeding. Extra-abdominal areas should also be examined. Cardiopulmonary examinations should be performed, since some intrathoracic diseases can show abdominal pain. A pelvic examination can be valuable in evaluating pelvic peritoneal in women experiencing abdominal pain (43). In men, a genitourinary examination should also be performed to evaluate the testicular torsion, trauma, hernia strangulated, or infections such as Fournier gangrene and STIs. The abdominal and back examinations are useful to examine the costovertebral angel tenderness and can be indicative of pyelonephritis or obstructive uropathy. Obviously, the total medical history, physical examination, and laboratory tests are inadequate to accurately diagnose the cause of acute abdominal pain, but they are more applicable to acute abdominal pain in urgent and non-urgent cases and can also justify the choice of additional imaging for a more accurate diagnosis (1).

### Laboratory tests

Laboratory tests should be requested by considering a specific clinical question. In patients with abdominal pain, tests checking for systemic diseases are clinically important. The most commonly used tests in the ED for patients with acute abdominal pain include complete blood count (CBC), blood urea nitrogen (BUN), creatinine (Cr), serum electrolytes, cardiac enzymes, liver function tests, and urine analysis. Other tests after history taking and physical examination and based on a specific diagnosis should be requested (44). Leukocytosis is commonly seen in appendicitis, mesenteric ischemia, small bowel obstruction and pancreatitis. There is considerable sensitivity but slight specificity. In some patients such as pregnant women, the elderly, consumers of steroid drugs, it should be noted that the diagnostic value of leukocytosis is reduced. C-reactive protein (CRP) is elevated as an inflammatory marker in response to infections and inflammation (such as Crohn’s disease or neoplasia), but it cannot be distinguished between immediate and non-urgent causes alone. Procalcitonin is a precursor to calcitonin and is primarily secreted by the thyroid and lung cells. It is not detectable in healthy subjects, and prothaloxytine is secreted by an endotoxin or inflammatory cytokine of these cells. Various studies have examined its diagnosis of appendicitis and intestinal ischemia (45–47). In cases such as mesenteric ischemia and appendicitis, the diagnostic value of D-dimer has been investigated, but no significant evidence has been found for it (48, 49). Intestinal fatty acid binding protein is specific to small intestinal epithelial cells, occurs in intestinal damage, and can be released in the blood. Intestinal ischemia has been indicated to be correlated with the rate and severity of ischemia (50–52). Both serum lipase and serum amylase increase in pancreatitis, but the serum lipase is elevated later than amylase serum and remains longer. In cases of gallstone pancreatitis, studies have indicated that alanine aminotransferase (ALT) has a high positive diagnostic value of 150 IU/L (53). Aspartate aminotransferase (AST) has also been demonstrated to have a predictive value of approximately ALT in detecting gallstone pancreatitis. Lactate, although not specific to abdominal pain, can help to assess the cause of illness in critically ill patients (54). In child-bearing women and those with abdominal pain unexplained, pregnancy testing should be taken. Increasing serum urea over serum creatinine suggests dehydration, sepsis, or bleeding (8). The prolonged prothrombin time, along with a reduction in the number of platelets, suggests a liver disease in which liver function tests are also expected to be difficult. Diabetic ketoacidosis and Edison’s disease may show themselves with acute abdomen that both blood glucose and electrolyte concentrations can help to diagnose them.

### Imaging methods

Immediately after clinical evaluation, there is a considerable doubt about the urgency of the patient’s condition (44). Clinical evaluation alone is not sufficiently accurate for a specific diagnosis, and imaging modalities can increase the accuracy of diagnosis (1, 55, 56). It should be noted, however, that imaging tests have false-positive and false-negative results, and in cases of high suspicion of a particular disease, it cannot be ruled out by referring to it alone.

#### • Simple radiography

In the past, plain radiography, owing to its low cost and availability, was used as the primary imaging modality for patients with abdominal pain. Certainly, with the more accessible alternative methods, its use has become controversial. It is suggested that plain radiography should be used only in cases of intestinal obstruction, perforation, foreign body ingestion or catheter location (54). Using plain radiographs in patients with peritoneal symptoms, upright radiograph can detect air below the aperture. In cases where the sensitivity of plain radiographs is not sufficient for a given condition, such as a small bowel obstruction, the physician should consider that the plain radiography is normal in the event of a misdiagnosis.

#### • Computed tomography (CT) scan

There are considerable sensitivity and specificity for several diseases causing abdominal pain. In cases where multiple diagnoses are commonly suspected, a very good diagnostic test is recommended. In stable patients whose pathology does not have a clear surgical procedure, but in differential diagnosis of the disease, they have important abdominal pathologies, CT scan is a good diagnostic test. We should consider the risk of future exposure to radiation in patients who are supposed to be CT. When using contrast, its cost / benefit should be measured (54). CT-angiography has high sensitivity and specificity in cases of mesenteric ischemia. The use of CT has increased in recent years, but its disadvantages include higher costs, latency in diagnosis with respect to waiting time for imaging, and risks such as contrast-induced allergy, contrast-induced nephropathy, and exposure to ionizing radiation (1, 3).

#### • Role of ultrasound in abdominal pain

A total of 5–10% of the complaints of patients referring to the emergency department constitute acute abdominal pain, and treatment based on physical examination and laboratory test results can lead to delayed treatment or inappropriate diagnosis (1). Furthermore, in many cases, the use of different imaging techniques, such as CT-Scan, in addition to unnecessary increases in costs can lead to adverse effects such as exposure to radiation (57–59).

In contrast, ultrasonography with broad availability, relatively low cost and absence of radiation, has a unique advantage over CT-Scan as an emergency diagnostic tool (60). Moreover, it is employed as an excellent screening and diagnostic method, which has been accessible and inexpensive as well as welcomed by emergency staff over the past decade (61). Obviously, the overall diagnostic power of the US is lower than that of CT scan. In one article, the sensitivity of 89% for CT scan and 70% for the US was reported in terms of identifing the causes of acute abdominal pain. Nevertheless, considering all different aspects, CT scan can be more useful with the highest diagnostic sensitivity in cases where the US is negative or inconclusive (1). Based on the findings, CT-Scan is a diagnostic aid for patients with stable hemodynamics or stabilized patients suspected of intra-abdominal injury; however, it is not a good choice for patients with unstable hemodynamics (62).

In general, the most common indications of abdominal ultrasound in acute ill patients are blunt trauma, acute abdominal pain and undifferentiated hypotension (63). However, US examination can be used for critical patients including shock state, multiple trauma, cardiac tamponade, intracranial pressure assessment; medicine and surgery including musculoskeletal injuries, eye trauma, external body, abdominal aortic aneurysm, aortic dissection, deep vein thrombosis, pulmonary system, abdomen, renal stone and colic and soft tissue; and procedures including peripheral nerve block, peripheral vein access, central vein access, lumbar puncture, nasogastric tube placement, endotracheal site evaluation (61). However, the usefulness of using this diagnostic tool varies for different clinical conditions. Table 1 shows the position of US as a diagnostic tool according to different clinical conditions. As this table shows, the sensitivity and specificity of the use of ultrasound in the diagnosis of diseases, in addition to the dependence on the skill of the person performing it, are influenced by the mode of disease; therefore, it is suggested that in addition to these two factors, it would be reasonable to consider positive (+LR) and negative (−LR) likelihood ratios in the interpretation of the results of emergency ultrasound (64). However, the diagnostic power of this tool, particularly in critical cases where the health team must take time-sensitive decisions, is unmistakable. Since, in many cases, the final accuracy in performing US by the radiologist is significantly different from an emergency medicine specialist (EMP), its use by an EMP may be beneficial in view of the overall reduction in the duration of the diagnostic process (65).

**Table 1:** The position of ultrasound as a diagnostic tool according to different clinical conditions

**Target of ultrasound**		**Sensitivity**	**Specificity**	**Reference**
**Testicular torsion**		94.0%	96.0%	(66)
**Ectopic pregnancy**		99.3%	38.0–74.0%	(67)
**Appendicitis**		100%	80.0–90.0%	(68, 69)
**Pelvic fracture**		26.0%	96.0%	(70)
**Abdominal aortic aneurysm**		96.3–99.0%	98.0–100%	(71, 72)
**Deep vein thrombosis**		32.0–46.0%	97.0–100%	(73)
**Vascular dissection^1^**		98.0%	NA	(74)
**FAST^2^**	**Blunt abdominal trauma**	50.0–95.4%	78.4–99.0%	(75–77)
	**Penetrating trauma (Stable patients)**	43.0%	100%	(78)
	**Pelvic fracture**	26.0–96.0%	96.0%	(70, 79)
**E-FAST^3^**	**Pneumothorax**	42.7–77.0%	99.2–99.8%	(64, 80–82)
	**Hemothorax**	92.0%	100%	(83)
**RUSH^4^**	**Hypovolemic**	100%	72.7–96.2%	(65, 84)
	**Cardiogenic**	60.0–90.0%	98.0–100%	(65, 84)
	**Obstructive**	90.9%	98.2%	(65)
	**Distributive**	72.7–75.0%	100%	(65, 84)
	**Mixed**	63.6%	98.2%	(65)

1 Assessed by B-flow ultrasound and including abdominal aorta, cardiac, vertebral, iliac, and femoral arteries

2 Focused Abdominal Sonography for Trauma

3 Extended-Focused Abdominal Sonography for Trauma

4 Rapid Ultrasound in Shock

## Discussion

**Figure 1:**
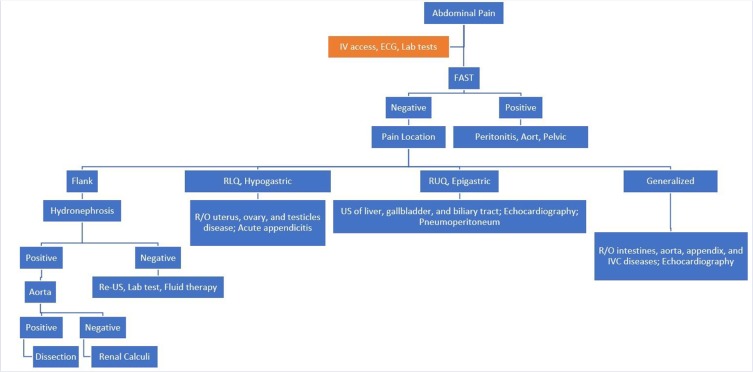
Diagnostic approached to a patient with acute abdominal pain using ultrasound in emergency department

Acute abdominal pain is one of the most common complaints of patients referring to the emergency department. Considering the wide range of causes causing multiple differential diagnoses, as well as the limited time of the health team in the emergency department for diagnostic and therapeutic measures, particularly in time-sensitive clinical conditions, ultrasound can be given by EMP as a diagnostic aid, which is considered to improve the overall diagnosis and treatment of patients, thereby reducing complications and complications. Based on the findings and reviewing the literatures, an algorithm has been prepared by the authors as a suggestion for diagnostic approached to a patient with acute abdominal pain using ultrasound in emergency department which is displayed in figure 1. Its performance definitely needs to be evaluated in further researches.

## Conclusions

Regarding the wide range of causes providing multiple differential diagnosis, as well as the limited time of the health team in the ED for diagnostic and therapeutic measures, particularly in time-sensitive clinical conditions, US offered by EMPs as a diagnostic aid is considered to improve the overall diagnosis and treatment of patients, thereby reducing complications.

## Appendix 1: Differential diagnosis of abdominal pain based on the pain location

**Table:**

**Location**	**Causes & Predisposing factors**
**Right upper quadrant**	Acute fatty liver Biliary dyskinesia Cholecystitis Cholelithiasis Cholangitis Colitis Diverticulitis Hepatitis Hepatic abscess Pulmonary embolism Pancreatitis Peptic ulcer disease Right lower lobe pneumonia Retrocecal appendicitis Sphincter of Oddi dysfunction Duodenal ulcer Fitzhugh-Curtis syndrome Nephrolithiasis Pyelonephritis
**Pregnancy**	HELLP syndrome Hepatic Rupture
**Epigastrium**	Peptic ulcer disease Cholecystitis Cholelithiasis Cholangitis Esophageal spasm Gastro-esophageal reflux Acute and chronic pancreatitis Pancreatic tumor Ruptured esophagus/Boerhaave’s syndrome Myocardial infarction Pericarditis Gastritis/dyspepsia Aortic dissection/rupture
**Left upper quadrant**	Esophagitis Gastritis Peptic ulcer disease Mesenteric ischemia Splenic infarction Splenic laceration Pericarditis Angina Myocardial infarction Pericarditis Pneumonia Pulmonary embolism Nephrolithiasis Pyelonephritis Aortic dissection Diaphragmatic hernia Acute and chronic pancreatitis Pancreatic tumor
**Right iliac fossa**	Appendicitis Inflammatory bowel disease Ovarian cyst/torsion Caecal diverticulitis Ileitis (e.g. Yersinia) Pelvic inflammatory disease/salpingitis
**Pregnancy**	Ectopic pregnancy Miscarriage
**Suprapubic**	Appendicitis Colitis diverticulitis Inflammatory bowel disease Irritative Bowel Syndrome Fibroids Ovarian mass/torsion Pelvic inflammatory disease Nephrolithiasis Pyelonephritis Cystitis/Urinary tract infection Urinary retention
**Pregnancy**	Miscarriage Placental abruption Uterine rupture
**Left iliac fossa**	Inflammatory bowel disease Ovarian cyst/torsion Diverticulitis Constipation Salpingitis Pelvic inflammatory disease
**Pregnancy**	Ectopic pregnancy Miscarriage
**Generalized abdominal pain**	Gastroenteritis Small/large bowel obstruction Mesenteric vein thrombosis Constipation Irritable bowel syndrome Ruptured viscus Boerhaave’s syndrome Intra-abdominal tuberculosis Mesenteric lymphangitis Mesenteric ischemia Peritonitis Narcotic withdrawal Sickle cell crisis Porphyria IBD Heavy metal poisoning Herpes zoster Muscle strain, hernia
**Other causes**	Diabetic ketoacidosis Hypercalcaemia Ingestion of foreign objects/caustic substances Lead poisoning Acute intermittent porphyria Addison’s disease Angioedema Testicular Torsion Enteric intussusception Abdominal migraine Systemic lupus erythematosus
